# Cannabis use amongst tinnitus patients: consumption patterns and attitudes

**DOI:** 10.1186/s40463-022-00603-8

**Published:** 2023-02-24

**Authors:** Dorsa Mavedatnia, Marc Levin, Jong Wook Lee, Amr F. Hamour, Kaye Dizon, Trung Le

**Affiliations:** 1 Faculty of Medicine, University of Ottawa, Ottawa, ON Canada; 2 Department of Otolaryngology - Head and Neck Surgery, University of Toronto, Toronto, ON Canada; 3 Sunnybrook Health Sciences Center, Toronto, ON Canada

**Keywords:** Cannabis, Tinnitus, Medical marijuana

## Abstract

**Background:**

Tinnitus has a significant impact on quality of life and causes considerable psychological distress. Cannabis is known to modulate neuron hyperexcitability, provide protection against auditory damage, and has been used for treatment for many diseases which have physiological similarities with tinnitus. The objective of this study was to survey patients presenting with tinnitus regarding their perspectives and usage patterns of cannabis.

**Methods:**

Patients with a primary presenting complaint of tinnitus in a tertiary neuro-otology clinic completed a 18-item questionnaire assessing perception, attitudes, and cannabis usage patterns.

**Results:**

Forty five patients completed the survey (mean age: 54.5 years, 31 females and 14 males). Overall, 96% of patients reported that they would consider cannabis as treatment for their tinnitus. Patients considered cannabis use for auditory symptoms (91%), and symptoms related to their tinnitus, such as emotional complaints (60%), sleep disturbances (64%), and functional disturbances (56%). 36% of patients had previously used cannabis and 22% of patients reported cannabis use at the time of the study. 80% of patients that were actively using cannabis reported that it helped with tinnitus-related symptoms, such as dizziness, anxiety, bodily pain, and sleep disturbances. Most patients would prefer to use edibles (62%), tablet (58%) and cream (47%) formulations of cannabis. Patients were concerned about the cost (29%), potential physical health implications (53%) and psychosocial side effects (60%) of cannabis. Over half of patients learned about cannabis from a friend or family member and only 22% of patients learned about cannabis from a physician or nurse.

**Conclusion:**

Cannabis use is common amongst patients with tinnitus and current users of cannabis reported that it helped with their symptoms. Most patients would consider its use as a potential treatment to alleviate their tinnitus-related symptoms and are interested in learning more regarding its use. By understanding how cannabis is perceived by tinnitus patients, healthcare providers can provide appropriate patient education.

**Graphical abstract:**

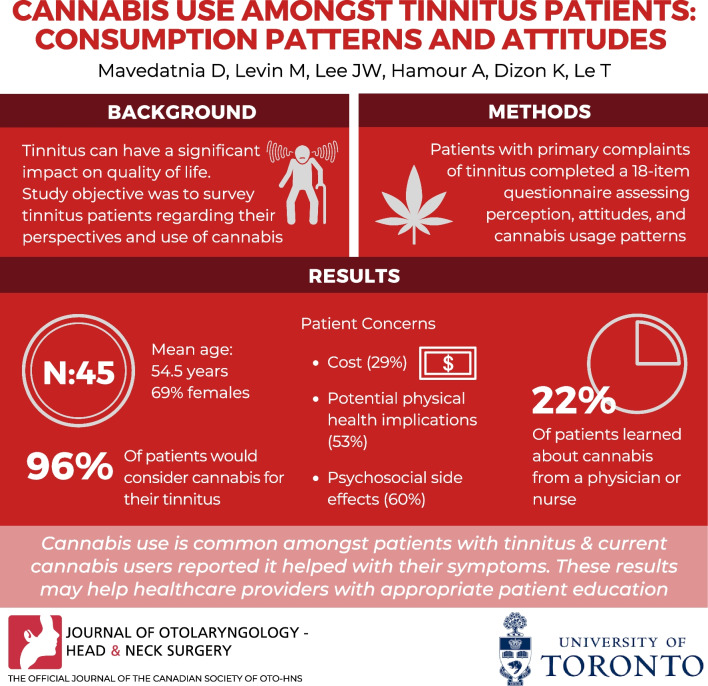

## Introduction

Tinnitus is the perception of sound in the absence of an acoustic stimulus. The majority of tinnitus is subjective and perceived sounds can manifest as ringing, buzzing, whirring, humming, static, and hissing [1]. Tinnitus is one of the most common and distressing neuro-otological conditions, affecting various aspects of life, such as sleep, concentration, and mood [2]. Tinnitus can negatively impact quality of life and persistent symptoms can be debilitating and cause considerable psychological distress [3–5].

Almost 50% of all patients presenting with neuro-otological disorders have psychiatric symptoms, such as anxiety and depression [6–11]. Moreover, overlap between neuro-otological and psychiatric conditions have been described, suggesting possibilities for shared treatment [12]. Management of tinnitus can be challenging, with the foundation of treatment consisting of masking strategies, hearing amplification, anti-anxiolytics, and cognitive behavioral therapy [13, 14]. However, despite these treatments, patients often experience persistent symptoms and more impactfully, an impaired quality of life [15–18].

Cannabis is one of the most commonly used drugs in North America, with close to half of Canadians aged 15 or older having reported using it [19]. Cannabinoids can modulate hyperexcitability, are involved in protection of auditory damage, neural processing in the auditory system, and in non-auditory circuits associated with tinnitus [20]. It has been used in treatment of neuropathic pain, anxiety, depression, headaches and seizures, all of which have similarities or associations with tinnitus [21–23]. With its legalization in various nations, it has been considered as a potential treatment for tinnitus, with several tinnitus sufferers turning to it as a possible remedy [20]. Limited research exists regarding the use of cannabis as a therapeutic agent among the tinnitus patient population.

The objective of this study was to survey patients presenting with tinnitus on their perspectives and usage patterns of cannabis. The results of this study will contribute to the understanding of the current use of cannabis in the tinnitus population and may help researchers understand how to focus future tinnitus and cannabis research.

## Methods

Institutional ethics review board approval was obtained from Sunnybrook Health Sciences Centre in Toronto, Ontario, Canada (REB #4932). A cross-sectional survey was performed using an 18-item questionnaire, designed by the research team and adapted from a similar study investigating cannabis use in head and neck cancer patients [24]. During a six month period, patients were randomly selected and recruited from an outpatient neuro-otology clinic of three practicing neurotologists via convenience sampling. Eligible adult patients included those who presented with a primary complaint of tinnitus. The questionnaires were voluntarily completed, and survey responses were anonymous. Written consent was obtained from all patients.

Demographic data regarding patient age and sex were collected. Survey items focused on attitudes towards the use of cannabis, preferred cannabis route of consumption, and past and current cannabis use (Appendix 1).

All statistical analyses were performed using Microsoft Excel. Frequency and percentages were computed for categorical variables and means were calculated for continuous variables.

## Results

Fifty-three patients were approached, and forty-five patients completed the questionnaire, achieving a 85% response rate. The median age was 56 years (range 31–76). There were 31 females (69%) and 14 males (31%).

### Patient cannabis use patterns

**Fig. 1:**
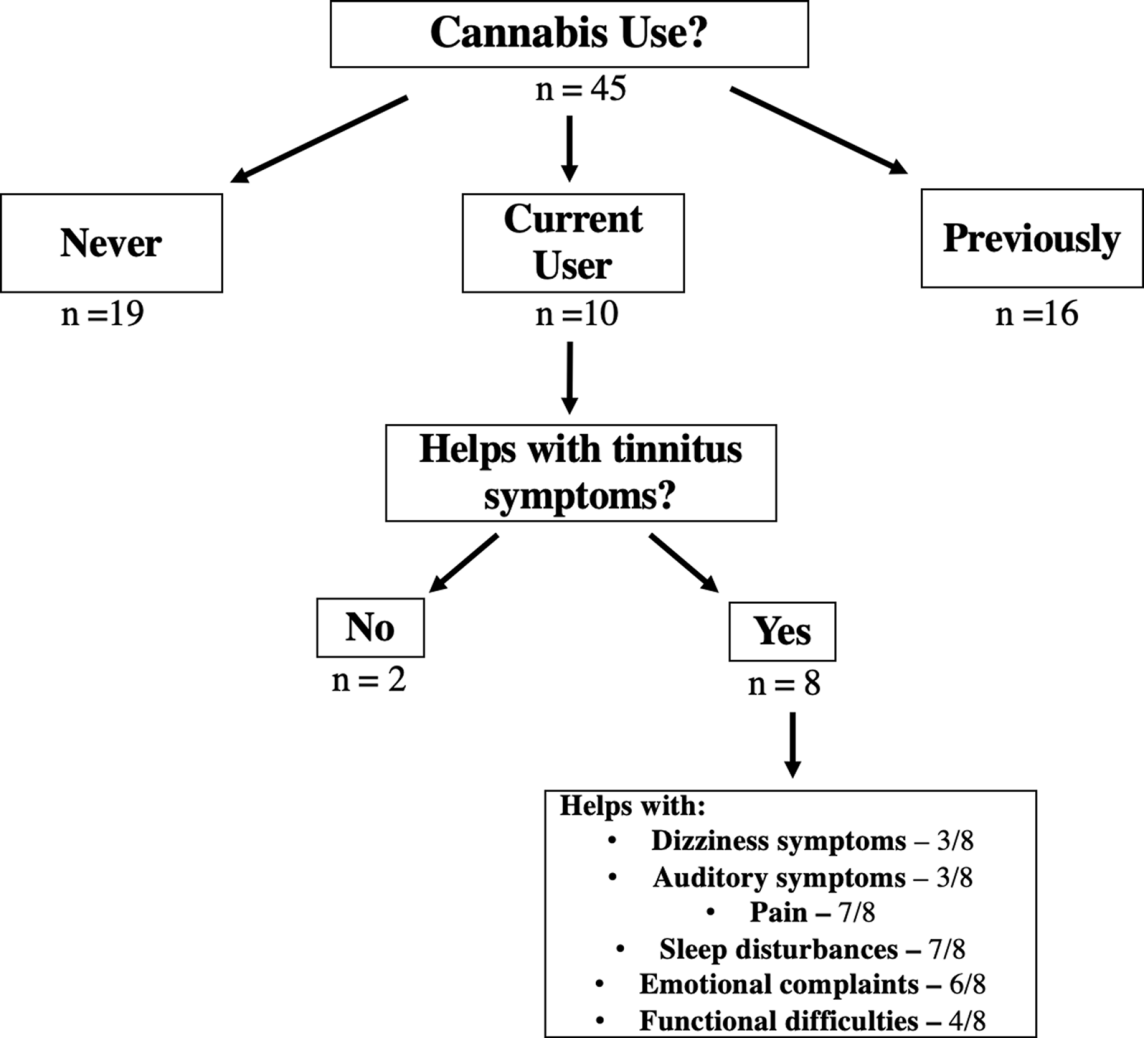
Cannabis use patterns and benefits

It was found that 42% (19/45) of patients had never used cannabis, 36% (16/45) previously used cannabis at some point in the past, and 22% (10/45) reported current cannabis use at the time of questionnaire completion (Fig. 1). Among patients that previously used cannabis, the most recent cannabis use ranged from 3 months to 50 years prior to completing the questionnaire. Two patients reported use only one time.

Among patients that were actively using cannabis (10/45), 6 patients were male and 4 were female, with age ranging from 31 to 70 years. Frequency of use ranged from five times per day to one time per month and duration of cannabis use ranged from two months to 50 years. Among these 10 patients, 6 patients reported using edibles (60%), 2 patients reported using tablets (20%), 6 patients reported smoking/vaporizing (60%), 3 patients reported using oil (30%), and 1 patient reported using cream (10%) (Table 1). Patients reported obtaining their cannabis from family members, medical cannabis stores, government stores, online, and local drug stores.

**Table 1:** Attitudes towards cannabis use formulations

	Total patients experiencing tinnitus (n, %) n = 45
**Preferred formulation of cannabis use**	
Edible	28 (62)
Tablet	26 (58)
Cream	21 (47)
Vaporizing	9 (20)
Oil	6 (13)
Smoking	3 (7)
Patch	1 (2)

	Patients currently consuming cannabis (n, %) n = 10
**Current formulation of cannabis use**	
Edibles	6 (60)
Smoking/vaporizing	6 (60)
Oil	3 (30)
Tablets	2 (20)
Cream	1 (10)

### Patient reported cannabis benefits

Of the 10 patients currently using cannabis, 80% (8/10) reported that it helps with tinnitus-related symptoms. Patients reported that it helped with dizziness/unsteadiness/disequilibrium (3/8, 38%), auditory symptoms (3/8, 38%), emotional difficulties (anxiety, depression, feeling upset, fear) (6/8, 75%), pain (headache, neck pain/aches) (7/8, 88%), sleep disturbances (7/8, 88%), and functional difficulties (concentration, fatigue, work disturbances) (4/8, 50%) (Fig. 1).

### Patient reported attitudes towards cannabis use

Overall, 96% (43/45) of patients responded that they would consider cannabis as a treatment for their tinnitus. Patients also considered cannabis as a viable treatment for their tinnitus-related symptoms (Table 2).

**Table 2:** Attitudes towards cannabis use for tinnitus-related symptoms

	Total patients experiencing tinnitus (n, %) n = 45
**Consider cannabis as treatment for tinnitus**	
Yes	43 (96)
No	2 (4)
**Tinnitus-related symptoms to consider cannabis use for**	
Auditory symptoms	41 (91)
Emotional complaints	27 (60)
Sleep disturbances	29 (64)
Pain	4 (9)
Functional disturbances	25 (56)

If patients were to use cannabis-derived medications, they would prefer to use edibles (62%, 28/45), tablets (58%, 26/45), cream (47%, 21/45), vaporizing (20%, 9/45), oil (13%, 6/45), smoking (7%, 3/45), and the patch (2%, 1/45) as routes of delivery (Table 1).

The recent legalization of cannabis in Canada made 73% (33/45) of patients somewhat-to-much more likely to use cannabis whereas 27% (12/45) of patients did not feel that this impacted their likelihood of using cannabis.

It was found that 51% (23/45) of patients learned about cannabis from a friend or family member, 22% (11/50) from social media, 22% (10/45) from a doctor or nurse, 20% (9/45) from a website or blogs, and 22% (10/45) received no information regarding cannabis. Almost all (98%, 44/45) patients were interested in learning more about cannabis if it were shown to help with tinnitus-related conditions. The most commonly listed professionals that patients wanted to receive this information from was their physician (36/45, 80%). Patients would also seek information regarding cannabis from nurses (3/45, 7%), cannabis clinics or stores (2/45, 4%), online sources (2/45, 4%), and pharmacists (1/45, 2%).

### Patient reported cannabis concerns

Patients were concerned about the cost (29%, 13/45), physical health (53%, 24/45), and psychosocial (60%, 27/45) side effects of cannabis.

## Discussion

This is the first study to assess perspectives and usage patterns of cannabis in patients experiencing tinnitus. The results of this study demonstrate an active interest amongst patients with tinnitus to consider cannabis as a potential adjunctive treatment for symptom management. Moreover, cannabis use is both common and can be beneficial in this patient population. An understanding of patient attitudes towards cannabis use is a prerequisite to exploring its potential use in clinical practice.

One theory of tinnitus is that of neuronal hyperexcitability in the auditory brain region [25]. Believed to be initially triggered by trauma, damaged auditory nerve fibers undergo maladaptive neural plasticity, which leads to decreases in inhibition and increases in excitation of multiple regions of the peripheral and central auditory pathway. This leads to an imbalance that ultimately causes neural hyperexcitability and aberrant activity that creates a false sensation of sound that is perceived as tinnitus [25]. Antiepileptic drugs, such as lamotrigine and gabapentin, have been studied as pharmacological treatment for tinnitus, given their inhibitory effect in the central nervous system [26–28]. However, there is insufficient evidence to support the use of antiepileptic drugs for tinnitus and it has not shown to be beneficial compared to placebo [28].

Endocannabinoid receptors are expressed in the vestibular nucleus complex (VNC) and have been theorized to suppress abnormal neuronal activity, inhibit neurotransmitter release, and play an autoregulatory role [29]. Activity of cannabinoid 1 (CB1) receptors may guard against neuronal hyperexcitability, having been shown to suppress epileptiform and seizure activity in animals [25, 30, 31]. Through other pathways, cannabinoids have been shown to possess anti-inflammatory, antiemetic, anxiolytic, sedative, and antioxidant properties [32–34]. Furthermore, early models of tinnitus have similarities with neurological disorders, such as neuropathic pain and epilepsy, both of which can be modulated by cannabinoids [20, 22, 35–37]. Given that cannabinoids possess neuroprotective effects in the cochlea and can modulate neuroinflammatory responses in the auditory system, cannabis may be a novel pharmacological candidate for treatment of tinnitus [20, 38, 39].

However, the literature is divided on the impact of cannabis on tinnitus as studies have found opposing results. Only two studies to date have found associations with tinnitus as a cannabis-related side effect [40]. In animal studies, cannabinoids were found to increase tinnitus in rat models, and although it was found to be otoprotective, but it was not effective in reducing tinnitus in guinea pigs [41, 42]. In human studies, there are contrasting results in the association between cannabis and tinnitus. In one study, tinnitus was found to have no association with cannabis use, while another study found a correlation between tinnitus and cannabis use, but not frequency of use or tinnitus severity [43, 44]. Studies have also found that cannabis use can worsen or induce tinnitus [43–46]. However, causative conclusions cannot be drawn from these studies as they are mainly correlative in nature. If an association exists between cannabis and tinnitus, there are three possible directions: (1) the experience of tinnitus increases cannabis use, (2) cannabis use increases tinnitus symptoms, (3) an extrinsic factor increases both variables [44]. Mood disorders, a possible contributing extrinsic factor, have been shown to increase both cannabis use and tinnitus perception [12, 47]. The relationship between tinnitus and cannabis is complex and likely multifactorial, influenced by psychological factors, drug formulation, administration route, and concetration [20, 44]. It is possible that patients experiencing tinnitus rely on cannabis as a form of self-medication. Given the lack of high quality prospective research on the effect of cannabis on tinnitus, the available evidence can neither support nor refute its use. Further research is needed to explore the role of cannabis in tinnitus to guide therapeutic interventions.

Over 95% of patients in this study reported that they would consider cannabis as treatment for their tinnitus and its associated symptoms. Patients’ willingness to consider alternative therapies, such as cannabis, for tinnitus can be due to several reasons. Firstly, tinnitus can be exceedingly burdensome as it is associated with insomnia, irritability, concentration difficulties, interruptions in daily activities, and psychiatric symptoms such as anxiety and depression [16, 48]. Its consistent presence and the lack of control that patients experience results in varying emotional impacts, ranging from mild irritation, to anxiety, depression, insomnia, and even suicide [35]. Secondly, patients also often do not receive adequate symptom relief from conventional therapy [16–18]. Even conventional therapy, such as sound masking, may not be preferrable for patients given the cost associated with hearing aids and the introduction of additional noise stimulus that might not be much different than a patient’s tinnitus. Cognitive behavioral therapy is also not widely available or adequately funded by insurance or a public health system.

Antidepressants, anti-anxiolytics, and cognitive behavioral therapy are current treatments for tinnitus, with the latter being the best-established treatment to date [13]. This suggests that tinnitus may be maintained and influenced by psychological and cognitive factors. Psychiatric conditions may be the primary cause, or co-exist with neuro-otologic conditions [48]. Cannabis use is common in patients with mood and anxiety disorders and it has efficacy in reducing anxiety behaviors in patients with generalized anxiety disorder, panic disorder, and social anxiety, without producing anxiogenic effects [49]. Pharmacological treatment of mood disorders with anxiolytics and antidepressants resulted in a reduction in tinnitus symptoms [12, 49, 50]. Given the association of tinnitus with anxiety, depression, migraines, and insomnia, coupled with the therapeutic efficacy of cannabis in managing aforementioned symptoms, it is reasonable to consider that patients with tinnitus may also benefit from cannabis treatment.

In this study, sleep disturbances, emotional difficulties, such as anxiety, depression, and fear, and pain were the most frequently reported symptoms that patients would consider cannabis for. They were also the most reported tinnitus-related symptoms that improved with cannabis use. The overlap between these two entities highlights that psychological symptoms are a primary source of distress for patients with tinnitus and that patients are eager to alleviate these symptoms. Cannabis may an attractive therapeutic for these patients as when used for depression and anxiety, it also led to a decrease in pain, and improved quality of life and sleep [50].

Patients in this study were mostly likely to consider edibles, tablets, and cream if they were to use cannabis. However, smoking/vaporizing was one of the most commonly used routes of delivery. There appears to be a level of discordance between the preferred and actual method of cannabis use. Similar results were seen in a survey that assessed cannabis use amongst head and neck cancer patients [24]. The disconnect may be explained by concern of cost and physical health side effects of cannabis, which were reported in 29% and 53% of patients, respectively. The cost of oil, edible, tablet forms of cannabis are greater compared to the smoking and vaporizing forms, with the latter also being known to cause respiratory consequences.

This study found that 73% of patients were somewhat-to-much-more likely to consider cannabis use after its legalization in Canada in 2018. Previous research has shown that those who find a behavior socially acceptable are more likely to engage in it [51]. Similarly, the social acceptability of cannabis is higher in individuals that report anxiety or acute pain [52]. Adults with medical conditions have a high prevalence of cannabis use compared to adults without medical conditions, and are more likely to report cannabis use for medical reasons [53]. The primary concern for patients were the possible psychological side effects of cannabis, such as psychosis, paranoia, and social impairment. Given that patients with tinnitus may be increasingly debating or engaging in cannabis use, physicians can consider discussing cannabis’ safety profile and exploring patients’ concerns.

Half of the patients in this study received information regarding cannabis from a family member or friend, despite the majority of patients stating that they wish to receive information from a physician. Only 20% received information about cannabis from a healthcare professional. Individuals that receive most of their information regarding cannabis from social media, internet, or friends and relatives are more likely to believe unsupported claims about cannabis [54]. Given that 96% of respondents were interested in learning more about cannabis if it were shown to improve tinnitus, it is imperative that public health campaigns and otolaryngologists managing patients with tinnitus are the primary source of information for patients to ensure they are receiving accurate information.

This study has several limitations. The duration or severity of patients’ tinnitus was not captured as part of the survey and may have played a role in influencing motivation to use cannabis. A validated tinnitus scale, such as the Tinnitus Handicap Inventory, may have been helpful in quantifying tinnitus symptom severity. Many patients noted in the questionnaire that reasons for considering cannabis treatment were due to limited relief of symptoms from current treatments. Although symptom duration in patients experiencing tinnitus was not found to be associated with quality of life, it is possible that it may affect their perception of cannabis as a treatment option to manage their symptoms [15]. Furthermore, convenience sampling was utilized, which may have limited the representativeness of this tinnitus patient population. In addition, this study is subject to recall bias. Patients may not have been able to accurately recall whether or not they previously consumed cannabis, potentially leading to an underreporting of cannabis use pattern rates.

## Conclusion

Cannabis use is common amongst patients with tinnitus and most participants would consider cannabis as a treatment option to manage their symptoms. Almost all patients were interested in learning more about cannabis if proven to help with symptoms of tinnitus, but physicians must be aware that most patients receive their information on cannabis from non-medical sources. This data may lay the groundwork for future research and clinical trials on cannabis use for tinnitus alleviation. Otolaryngologists can develop an understanding of patient attitudes and usage patterns to guide patient counseling on the use of cannabis for symptoms associated with tinnitus.

## Data Availability

Not applicable.

## Appendix 1

1. **Would you consider cannabis-derived medications (CBD, THC, etc.) as treatment for your tinnitus/dizziness?**
  (A) Yes
  (B) No

2. **Would you consider cannabis-derived medications (CBD, THC, etc.) as treatment for the following dizziness-related symptoms (if applicable): (Check all that apply)**
  (A) Dizziness/unsteadiness/disequilibrium
  (B) Functional difficulties (concentration, fatigue, work disturbances)
  (C) Emotional complaints (anxiety, feeling upset, depression, fear)
  (D) Pain (headache, neck pain/aches)
  (E) None

3. **Would you consider cannabis-derived medications (CBD, THC, etc.) as treatment for the following tinnitus-related symptoms (if applicable): (Check all that apply)**
  (A) Auditory symptoms (I.E. ringing, clicking, hissing)
  (B) Functional difficulties (concentration, fatigue, work disturbances)
  (C) Emotional complaints (anxiety, feeling upset, depression)
  (D) Sleep disturbances (sleep initiation, poor quality of sleep)
  (E) None

4. **What are your reasons for considering cannabis treatment (if applicable)?**

5. **What concerns you about use of cannabis-derived medications? (Check all that apply)**
  (A) Cost
  (B) Physical health side effects (such as lung disease)
  (C) Psychosocial side effects (such as psychosis, paranoia, social impairment etc.)
  (D) Other:

6. **What route of delivery would you prefer, if you were to use cannabis derived medications? (Check all that apply)**
  (A) Smoking
  (B) Vapourizing
  (C) Edible (in food or drink)
  (D) Tablet
  (E) Cream
  (F) Other:

7. **Where have you learned about cannabis? (Check all that apply.)**
  (A) I haven’t received this information
  (B) Medicinal cannabis store
  (C) Doctor or nurse
  (D) Recreational cannabis store
  (E) Friend / family member
  (F) Pamphlet or handout
  (G) Nutritionist
  (H) Newspaper / magazine article
  (I) Naturopath / herbalist
  (J) TV / radio advertisement
  (K) Social media (Facebook, Twitter, etc.)
  (L) Websites or blogs
  (M) Other

8. **If cannabis-derived medications were shown to help with tinnitus/dizziness-related conditions, would you be interested in learning more? (Check answer)**
  (A) Yes
  (B) No

9. **Who would you seek out to learn more about cannabis from? (Answer in space below)**

10. **Does the legalization of cannabis use in Canada make you more likely to use cannabis?(Circle number)**

11. **Have you previously used cannabis? (Check answer)**
  (A) Yes
    - If YES, when was the last time you used cannabis?
  (B) No

12. **Are you currently using cannabis?**
  (A) Yes
  (B) No

13. **How long have you used cannabis for? (Answer in space below)**
  - ____ years/months/weeks (circle applicable)

14. **How often do you use cannabis?**
  - ____ times per ____ year/month/week/day (circle applicable)

15. **Where do you get your cannabis? (Answer in space below)**

16. **If you use cannabis, in what form are you using it? (Check all that apply)**
  (A) Smoking
  (B) Vapourizing
  (C) Edible (in food or drink)
  (D) Tablet
  (E) Cream
  (F) Other (Answer in space below)

17. **If you use cannabis, do you feel that it helps with your tinnitus/dizziness-related symptoms?**
  (A) Yes
  (B) No

18. **If you answered YES to Question #17, please specific which symptoms cannabis is helping you with? (Check all that apply)**
  (A) Dizziness/unsteadiness/disequilibrium
  (B) Functional difficulties (concentration, fatigue, work disturbances)
  (C) Emotional complaints (anxiety, feeling upset, depression, fear)
  (D) Pain (headache, neck pain/aches)
  (E) Auditory symptoms (I.E. ringing, clicking, hissing)
  (F) Sleep disturbances (sleep initiation, poor quality of sleep)
  (G) Other (Answer in space below)
